# Galvanic vestibular stimulation down-regulated NMDA receptors in vestibular nucleus of PD model

**DOI:** 10.1038/s41598-022-20876-3

**Published:** 2022-11-08

**Authors:** Gyutae Kim, Nara Shin, Kyu-Sung Kim

**Affiliations:** 1 Research Institute for Aerospace Medicine, Inha University, Incheon, Korea; 2 Department of Otolaryngology Head & Neck Surg., Inha University Hospital, Incheon, Korea

**Keywords:** Molecular biology, Neuroscience, Biomarkers, Medical research, Engineering, Nanoscience and technology

## Abstract

Parkinsonian symptoms relief by electrical stimulation is constructed by modulating neural network activity, and Galvanic vestibular stimulation (GVS) is known to affect the neural activity for motor control by activating the vestibular afferents. However, its underlying mechanism is still elusive. Due to the tight link from the peripheral vestibular organ to vestibular nucleus (VN), the effect by GVS was investigated to understand the neural mechanism. Using Sprague Dawley (SD) rats, behavioral response, extracellular neural recording, and immunohistochemistry in VN were conducted before and after the construction of Parkinson’s disease (PD) model. Animals’ locomotion was tested using rota-rod, and single extracellular neuronal activity was recorded in VN. The immunohistochemistry detected AMPA and NMDA receptors in VN to assess the effects by different amounts of electrical charge (0.018, 0.09, and 0.18 coulombs) as well as normal and PD with no GVS. All PD models showed the motor impairment, and the loss of TH^+^ neurons in medial forebrain bundle (mfb) and striatum was observed. Sixty-five neuronal extracellular activities (32 canal & 33 otolith) were recorded, but no significant difference in the resting firing rates and the kinetic responding gain were found in the PD models. On the other hand, the numbers of AMPA and NMDA receptors increased after the construction of PD model, and the effect by GVS was significantly evident in the change of NMDA receptors (*p* < 0.018). In conclusion, the increased glutamate receptors in PD models were down-regulated by GVS, and the plastic modulation mainly occurred through NMDA receptor in VN.

## Introduction

Pathological evidence demonstrates that the loss of dopaminergic neuron in the substantia nigra (SN) causes Parkinson’s disease (PD), which shows the progressive and neurodegenerative motor and non-motor symptoms^1–3^. Despite its irreversible functional regression, the PD-induced symptoms have been managed by electrical stimulation (deep brain stimulation, DBS) on specific brain regions, such as the ventralis intermedius nucleus of the thalamus (Vim), the posteroventral portion of the internal segment of the globus pallidus (GPi), and the subthalamic nucleus (STN)^4^. Each region was targeted for specific symptom. For instance, DBS on Vim was generally selected to subside the uncontrolled tremors, and the stimulation was applied on GPi and STN to reduce dyskinesia^3,5–7^. Despite the well-defined methodological application and therapeutic benefits, the underlying mechanism of DBS is still elusive^3,8–10^. Some circuitopathic hypotheses suggested that the electrical stimulation counteracted to block the neural activity around the implemented electrode, and the activity was replaced with the applied stimulation^11,12^. A recent study demonstrated that DBS compensated for the oppositely generated theta and the beta signals in the posterior-dorso-lateral oscillatory (motor) region of the subthalamic nucleus during tremor. This study also suggested that an adaptive DBS might be an effective stimulus for the reduction of tremor^13^. However, any proposed hypotheses rarely revealed the exact role of the electrical stimulation as an inhibitory or an excitatory signal in the complex neural network. Apparently, DBS worked as a controversial role in GPi and STN; DBS inhibited local neuronal units while the same stimulation excited the efferent neurons, affecting thalamus and GPi, respectively^3^. Thus, a simplified circuitopathy caused by an electrical stimulation was necessary to interpret the mechanism of the stimulation.

Vestibular system is a highly plastic system, and its plastic mediation is built by the molecular and cellular modification^14–17^. The modified responses are mainly regulated by various types of neurotransmitters, and the glutamate is one of excitatory neurotransmitters, which is known as the most abundant amino acid in the brain^18,19^. The glutamate was transmitted through its relevant receptors, and these receptors were divided into two subtypes, ionotropic type as ligand-gated cation channels and metabotropic type as G-protein coupled receptors^19^. Ionotropic receptors directly involved the transmission of glutamate while metabotropic receptors worked through the second messenger to ion channels. The ionotropic receptors are again subdivided into three receptors, such as α-amino-3-hydroxyl-5-methyl-4-isoxazole-propionic acid (AMPA), N-methyl-D-aspartate (NMDA), and 2-carboxy-3-carboxymethyl-4- isopropenylpyrrolidine (kainate)^19^. Generally, NMDA receptor is known to govern the neural plasticity while AMPA receptor is considered as the regulator in the transmission of the glutamate^16,20^. During the plastic responses by NMDA receptors, their number and structure were rapidly regulated^21^. Thus, the changes in the number and structure of NMDA receptors might indicate the plastic activity under a given circumstance.

Galvanic vestibular stimulation (GVS) is known to relieve some motor disabilities, which directly activates the vestibular afferent neurons^22–24^. The generated signals are then transmitted to the vestibular nucleus, which is one of the core nuclei converging the incoming sensory information^25,14^. Using the conveyed information by GVS, various affirmative effects were observed in the Parkinson’s disease patients; the improvements in the anterior bending posture^26^, in upper and lower extremities^27^, and in body sway^28^. However, the clinical evidence had also indefinable neurophysiological gaps to understand the mechanism from the electrical stimulation to the tuning motor control. Here, we investigated the regulation of glutamate receptors by GVS to understand the interrelation between electrical stimulation and NMDA receptors in Parkinson’s disease, focusing on AMPA and NMDA receptors in vestibular nucleus (VN). Based on their relation, the motor recovery by GVS would be described in Parkinson’s disease.

## Results

**Figure 1:**
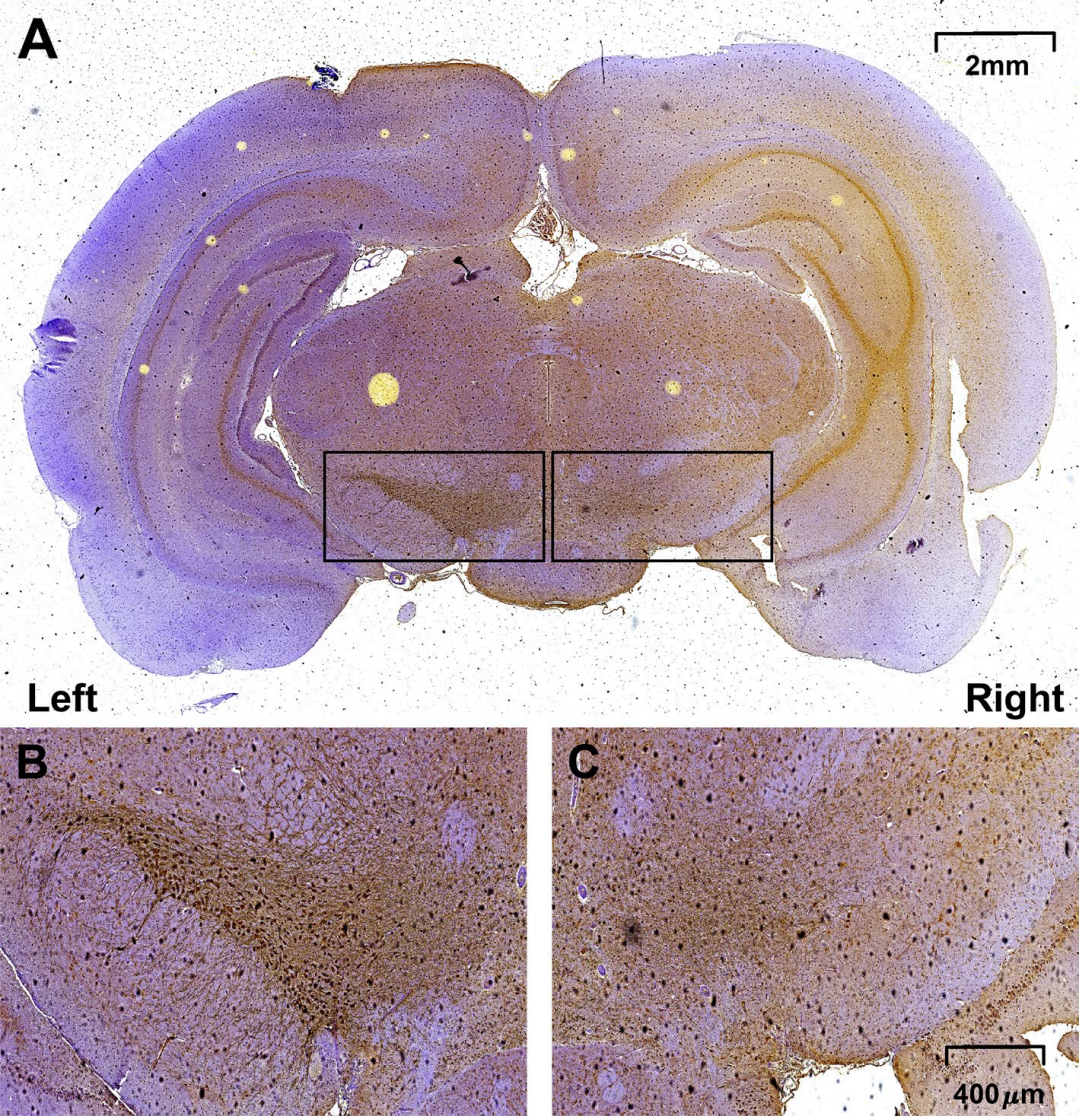
Loss of TH^+^ neurons in medial forebrain bundle (mfb) after the injection of 6-OHDA. The injection in the right side reduces TH^+^ neurons while the left side maintains its concentration.

**Figure 2:**
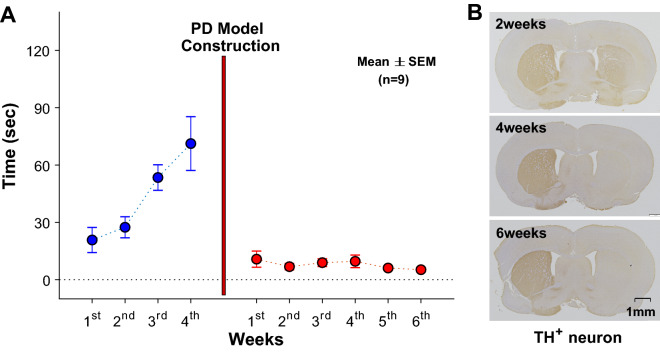
Locomotion activity in time (sec) (**A**) and loss of TH^+^ neurons in striatum (STR) in 2, 4, and 6 weeks after the injection of 6-OHDA (**B**). The performance of locomotion increases, but it decreases after the model construction. The immunoreaction to 6-OHDA in STR shows the TH^+^ neurons are reduced depending on the time elapses (2, 4, and 6 weeks). Again, the decreased TH^+^ neurons are observed only in the right side of 6-OHDA injection. (blue & red circles for normal animals and PD models, respectively).

Following the injection of 6-OHDA, the selected PD models were used to assess the loss of TH^+^ neurons in mfb (n = 1) (Fig. 1) and striatum (STR) (n = 3) (Fig. 2). As the toxic injection was made at right side based on the sagittal line, all TH^+^ cellular losses occurred at the right side (see **Parkinson’s Disease model**). The immunoreaction to 6-OHDA in mfb was examined at 1 week after the injection. As shown in Fig. 1, the TH^+^ neurons in mfb decreased more at the right while those in the left side had few changes, indicating that the toxic injection into mfb directly affected the cellular reduction. The similar consequence was observed in STR, depending on the elapse times (2, 4, and 6 weeks) (Fig. 2B). As time went after the toxic injection, the TH^+^ neurons in STR decreased, and those after 4 and 6 weeks were barely observed at the right as compared with those at the left side.

The behavioral activity based on rota-rod test (n = 9) showed the decline in the performance of locomotion after the model construction (Fig. 2). Before the toxic injection for PD model, an animal generally increased the averaged (± standard error of mean, SEM) time on the rolling rod from 20.78 (± 6.59) to 71.23 (± 14.06) seconds (blue circles). However, the performing time dramatically dropped by 5.24 (± 1.23) seconds (red circles), indicating the reduced locomotion after PD model construction. According to the obtained results in the immunoreaction to TH^+^ neurons and the behavioral activity on the rota-rod, the toxic injection successfully constructed PD model, providing cellular and functional declines.

**Figure 3:**
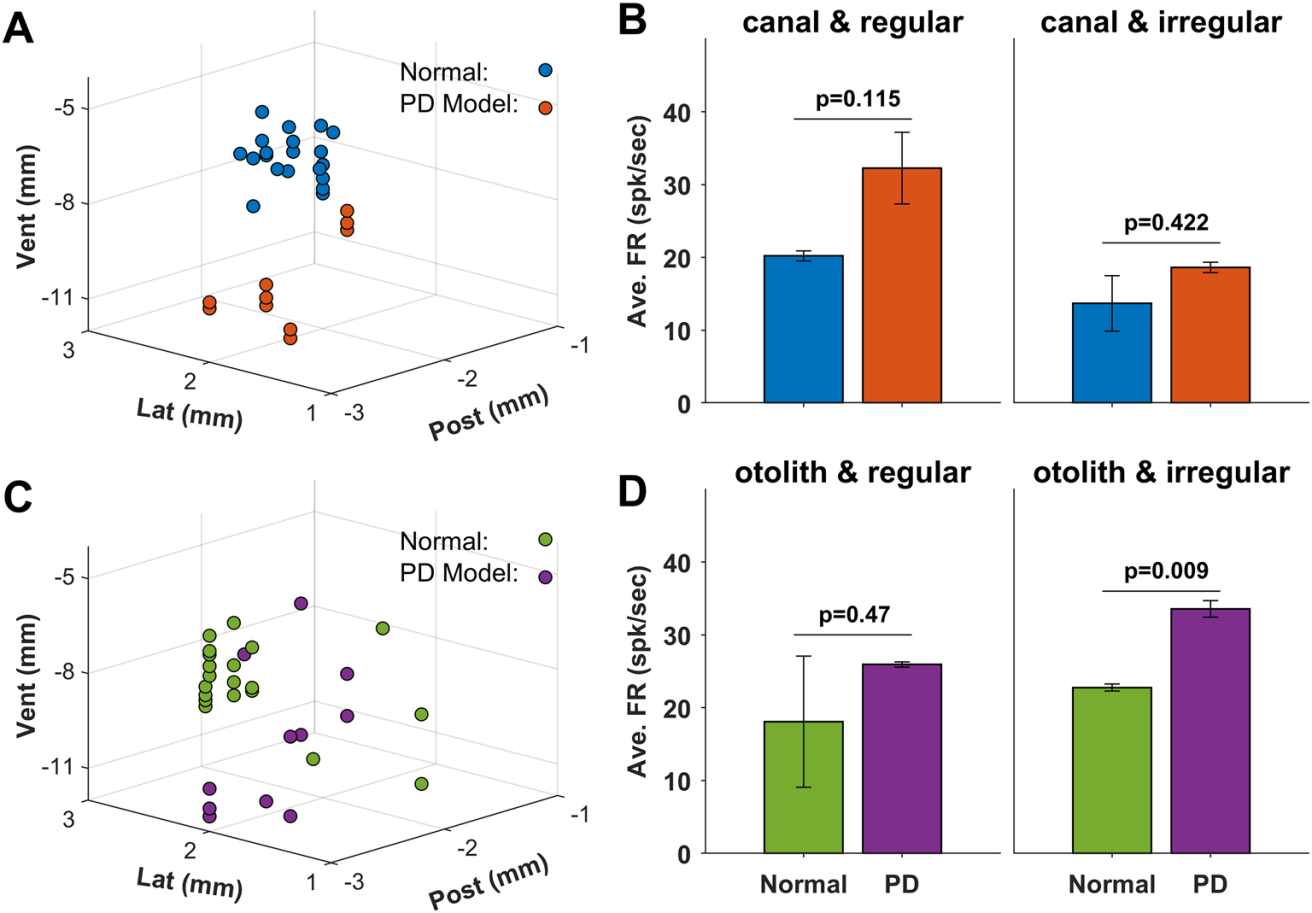
Extracellular neural recording position (**A** & **C**) and the population of the averaged firing rates (FR) for canal (**B**) and otolith (**D**) from normal (blue & green) and PD (orange & purple). Each group is subdivided into the regular and the irregular units based on the discharge regularity. Except the otolith and irregular group, all average FRs increased after the construction of PD with no statistical significance (*p* > 0.115).

**Figure 4:**
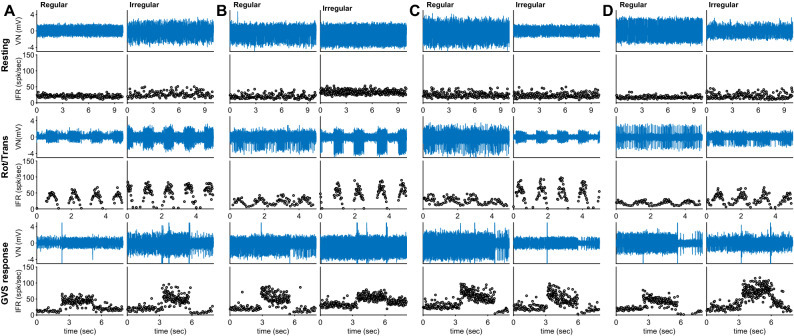
Examples of extracellular neuronal activity of canal-related in normal (**A**) and PD model (**B**), and those of otolith-related neurons in normal (**C**) and PD (**D**). Each group was subdivided based on the discharge regularity. Each row represents a neuronal example, and the neuronal spikes and its relevant instantaneous firing rate (IFR) are shown during resting period, rotation/transmission, and GVS.

Using 23 animals, extracellular neural recording was conducted. Of these animals, 18 animals had no toxic injection, five animals were used as PD models. Total 65 neuronal activities were recorded, and their specific recording locations were presented (Fig. 3A and C). Thirty-two activities were obtained from the canal-related neurons, and the others (33 activities) were the neuronal activities related with otolithic units. In the canal-related activity, two-third (62.5%, 20/32) activities were recorded from the animals with no toxic injection, and the similar population (63.6%, 21/33) was normal in the otolith-related activities. These activities were again divided into the regular and the irregular activities based on the discharge regularity (see **Analysis of Neuronal Signals**). The relevant examples presented in Fig. 4. Each example was tested based on no external stimulation (resting duration), the head horizontal rotation for canal-related neurons, the head linear transmission for otolith-related neurons, and 100 μA GVS. The normal canal and regular example maintained an IFR of 21.03 spk/sec during the resting, and its responding IFR reached to 49.12 spk/sec during the horizontal head rotation. The example was tonic reaching to 45.3 spk/sec during GVS. On the other hand, the normal canal and irregular example showed a lower IFR (18.29 spk/sec) than the normal canal and regular example, but its maximum IFR was 70.01 spk/sec during the horizontal head rotation, indicating that the responding IFR was higher in irregular than in the regular example. In addition, the response to GVS was phasic, decreasing from 66.12 to 36.20 spk/sec (Fig. 4A). In the neuronal canal examples of PD models, the IFRs of regular and irregular were 20.20 and 33.64 spk/sec, respectively. The rotational response of the regular example was smaller (ranged between 12.20 and 31.11 spk/sec) than that of the irregular neuron (up to 66.23 spk/sec). Unlike the normal examples, the IFR of regular example was phasic (ranged from 49.88 to 74.07 spk/sec), and that (58.91 spk/sec) of irregular example was tonic response to GVS (Fig. 4B). In the otolithic examples in the normal and PD models, the IFRs were similar as those of the canal examples during the resting (25.30, 23.61, 17.85, and 21.89 spk/sec for normal regular, normal irregular, PD regular, and PD irregular, respectively). Similarly observed in the canal-related neurons, the otolithic neuronal responses indicated that the irregular neurons of both normal and PD model were more sensitive to the kinetic stimulation (linear transmission) than the regular neurons. Three examples were phasic responses ranged from 27.89 to 77.82 spk/sec, and the otolithic irregular of PD model was tonic response to GVS (72.60 spk/sec) (Fig. 4C and D). Populational comparison among all categorized groups was conducted based on the resting IFR (Fig. 3B and D). Comparing between normal and PD model, IFR of PD models (18.63–33.56 spk/sec) was higher than that of the normal animals (13.68–22.76 spk/sec). However, statistical analysis revealed that there was no statistical significance (*p* > 0.115) except in the IFR from otolithic irregular neurons of PD models (*p* = 0.009).

**Figure 5:**
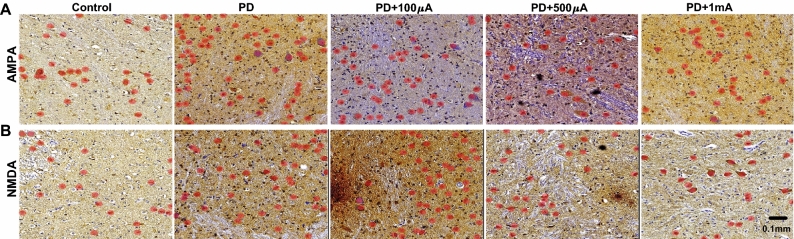
Immunohistochemistry examples with glutamate receptors in vestibular nucleus (VN) in control, PD, PD with 100 μA, PD with 500 μA, PD with 1 mA. The targeted receptors are AMPA (**A**) and NMDA (**B**), and the number of the stained receptors indicated the effect by GVS. The given strength of GVS was calculated for electrical charge by Eq. 2.

Further assessment was made based on the glutamate receptors in VN, which provided the core pathway of the most abundant neurotransmitter in the brain. The targeted area (VN) was stained with antibodies to the glutamate receptors, AMPA and NMDA receptors, and their numbers in the prepared images were counted. As shown in the images with the detected receptors, the glutamate receptors were identified and counted under different conditions, such as control, PD model, PD model with 100 μA, PD model with 500 μA, and PD model with 1000 μA (see **Immunohistochemistry (IHC) & Assessment of glutamate receptors**) (Fig. 5). The applied GVS in different strength was used to calculate its relevant electrical charges based on its conservation (Eq. 2).

**Figure 6:**
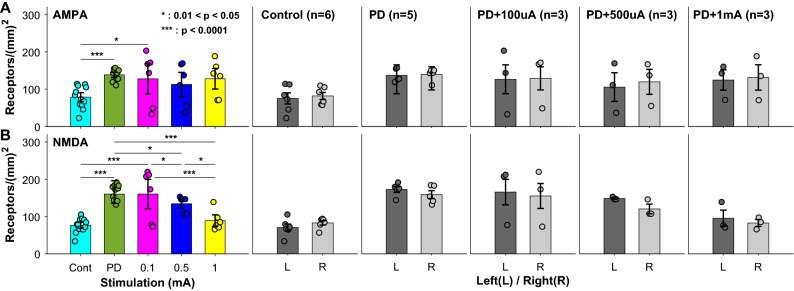
Populational distribution of AMPA (**A**) and NMDA glutamate receptors (**B**) depending on the electrical charges by GVS. Each column presents a total population of controls, PD, PD with 100, 500, and 1000 μA), and their comparisons between left (L) and right (R) are shown with no significance (*p* > 0.721 in AMPA & *p* > 0.096 in NMDA receptor). Both receptors increased significantly after the construction of PD model (*p* = 1.33 × 10^−5^ in AMPA & *p* = 1.85 × 10^−9^ in NMDA receptor). Increased electrical charge has no effect on the change of AMPA receptors while the NMDA receptors decrease as the electrical charge increases (*p* < 0.0175).

The populations were compared using 20 animals (six controls, five PD models, and three for PD models with different stimulation, such as 100, 500, and 1000 μA) (Fig. 6). Under each condition, the numbers of receptors in the left and the right VN were separately compared, but there was no statistical difference in both AMPA (*p* > 0.721) and NMDA receptors (*p* > 0.096). On the other hand, both receptors increased significantly after the construction of PD model, and the numbers in a unit area (mm^2^) were ranged from 78.58 ± 12.02 to 138.34 ± 7.22 in AMPA (*p* = 1.33 × 10^−5^) and 76.92 ± 7.92 to 165.86 ± 30.40 in NMDA (*p* = 1.85 × 10^−9^). However, the increased receptors were statistically maintained in AMPA receptors (*p* > 0.188) regardless of the applied electrical charge by GVS (Fig. 6A). In contrast, the relation between the number of receptors and the electrical charge was found in NMDA receptors responding to the applied GVS. After PD model construction, NMDA receptors decreased as the electrical charge increased (Fig. 6B). The numbers of NMDA receptors in the unit area of VN were 165.86 ± 30.40, 160.58 ± 39.24, 134.62 ± 12.14, and 89.21 ± 15.50 for PD model, PD with 100 μA, 500 μA, and 1 mA, respectively. Their relevant statistical tests indicated that NMDA receptors were not changed by GVS with 100 μA (*p* = 0.82), but there were significant decreases by GVS with 500 μA and 1 mA (*p* < 0.018). Thus, our current results suggested the change of glutamate receptors induced by GVS, and the NMDA receptor was the target protein affected by the electrical stimulation. Also, the required electrical charge was approximately more than 0.09 coulombs to modulate the number of the target receptor.

## Discussion

Electrical current-induced neuromodulation showed positive effects on the regulation of dysfunctional motor symptoms, and its application has been expanded to the neurological treatment and pain relief as well as neural plasticity^29–31^. Although the empirical consequences suggest the efficient regulation on the relevant symptoms, little underlying mechanism is still clear. Our study invested the neural responses at the molecular and the cellular levels in VN using Galvanic vestibular stimulation (GVS). In the neuronal responses, the firing rates (FR) and the kinetic gain were mainly examined, which provided the neural information related with the neural variability^32^ and the characteristic response to the head movement^33^. In the comparison between normal and PD model, the averaged resting FR and the kinetic of PD model were higher than those of normal animal, and the same results were observed no matter what regularity (regular vs. irregular) and origination (canal vs. otolith) (Fig. 3). This consequence matched to the change in the number of glutamate receptor with statistical significance (*p* = 1.33 × 10^−5^ & *p* = 1.85 × 10^−9^ for AMPA & NMDA receptor, respectively) (Fig. 6). Thus, the increased resting FR was possibly caused by the increased number of the glutamate receptors as suggested in some previous studies^34,35^. However, the GVS-induced effect on the glutamate receptors worked differently; the number of NMDA receptor decreased while that of AMPA receptor showed no significant change depending on the amount of electrical charge (Fig. 6). According to our results, the applied GVS affected the number of NMDA receptor, and the electrical charge of GVS was the core parameter for the modulation. Previous studies using GVS demonstrated the stimulation reduced the Parkinsonian symptoms, suggesting GVS regulated the neural information to correct the functional impairments^22–24,26–28^. The neural information under the impairments was modified by GVS, and this newly generated information improved the malfunctions. However, this empirical explanation was not enough to provide the insight for the underlying mechanism in the GVS-induced functional recovery.

The critical role of glutamate in PD was previously examined from the aspect of both pathophysiology and neurodegeneration^36–38^. In PD, the loss of dopamine reduced the dopaminergic information, and the glutaminergic information increased in the basal ganglia stimulating the dopamine release in STN^37^. At the same time, the enhanced glutaminergic signaling might damage the dopaminergic neurons causing the decrease of dopamine^37^. This secondary disturbance by the glutamate system aggravated the pathophysiological condition of PD^39^. Therefore, this compensatory cycle required a fine control of glutamate, and the activated glutamate receptors played the key role for the regulation of glutamate. In this study, the relation between GVS and glutamate receptors was demonstrated, indicating that the increasing electrical charge reduced the number of glutamate receptor. Especially, the response of glutamate receptor to GVS was found only in NMDA receptors while AMPA receptor was rarely affected by the stimulation (Fig. 6). As known, the glutamate induced an excitotoxic effect on the central nervous system, and its high extracellular concentration resulted in the depletion of antioxidant and oxidative glutamate toxicity. These consequences produced the cellular damages, reducing the cellular and the systemic functions^37,40^. In addition, the highly activated NMDA receptors increased the intracellular calcium (Ca^2+^) interrupting the function of cytoskeleton^41^.

The increased NMDA receptors indicated the neuronal depolarization in VN^42,43^. Following this estimation, the pathological indicator for the progress of PD was attained by the change of NMDA receptor, agreeing with previous evidence^44^. As the depolarization was characterized by the malfunctional activity of ion channels, the transmission of neural information was disturbed, and the electrical stimulation like GVS possibly led to a balanced concentration of some core neurotransmitters, such as different subtypes of glutamate. Especially, this modulation was conducted through NMDA receptor, and its number was controlled by the electrical charge by GVS as demonstrated in this study, agreeing with previous results^45,46^.

## Methods

Total 56 Sprague Dawley (SD, 275-562 g, males) were used in this study. Nine animals were used for behavioral responses after the construction of Parkinson’s disease (PD) model. Twenty-three and twenty-four animals were used for extracellular neural recording and immunohistochemistry, respectively. The animals were housed in a facility which stably maintained temperature (22–25 °C), humidity (40–60%), ventilation (10–15 times/hour), static pressure difference (> 5mmAq), and noise level (< 60 dB). In addition, a 12:12 h light–dark cycle was provided to the animals while they were housed. The animals were taken out of the housing facility only during an experiment, and they were re-housed in the facility as the experiment was completed. All procedures and principles of laboratory animal care were approved by the Animal Ethics Committee at Inha University (INHA 200,110–681). The current study was reported in accordance with ARRIVE guidelines, and all methods were performed in accordance with relevant guidelines and regulations.

### Parkinson’s disease model

The construction of a PD model was initiated by intraperitoneally injecting desipramine (12.5 mg/kg, Enzo, New York, US), which was known as a selective inhibitor of norepinephrine transporter for the restricted effects on dopaminergic neurons by 6-hydroxydopamine (6-OHDA, Sigma, St. Louis, US). By injecting the desipramine, thus, the induced toxicity by 6-OHDA affected a targeted lesion with a high selection. After the desipramine injection, the animal was anesthetized by an intramuscular injection of a mixed solution of Ketamine (1 µl/g) and Xylazine (0.33 µl/g). Once the animal was fully anesthetized, its head was fixed using a motorized stereotaxic apparatus (Neurostar, Tubingen, Germany). Then, its skull was surgically exposed to open a hole for 6-OHDA injection at a location (2 mm lateral and 2.5 mm posterior directions) away from Bregma. The target area of 6-OHDA injection was the medial forebrain bundles (mfb) at the right side, which was located ranged from 8.5 to 9.5 mm ventral direction from the hole. Using a 26-gauge syringe (Hamilton, Reno, US), 6-OHDA (8.0 μg/μL) was injected at the speed of 8 μL/min. Completed the toxic administration, the hole and the skull were sutured, and the animal had a rest at least for 1 week before its evaluation.

### Model evaluation

PD model evaluation was performed mainly by immunohistochemistry and rota-rod test. Due to the decrease of the positive tyrosine hydroxylase (TH^+^) in the substantia nigra (SN) induced by 6-OHDA administration, the immunohistochemistry was conducted to detect TH^+^. At the beginning, the whole brains were removed and fixated in a formalin solution with 10% neutral buffer (Sigma, St. Louis, US). After completing the fixation, the area containing SN was obtained from the brains, and its dissection was performed (slice thickness: 2.5–3 μm). Then, the prepared sections were stained using the primary antibody of Rabbit anti-Tyrosine hydroxylase (MilliporeSigma, Burlington, US), and they were incubated overnight at 4 °C. The stained sections were imaged by a motorized microscope (Olympus, Tokyo, Japan), and the captured images were stored in a magnification of 400 times (ocular: × 10 and objective: × 40) of the actual size. For the comparison of the affected with the unaffected area, the detected TH^+^ was estimated by its different density by counting the stained target neurons.

### Extracellular neural recording & Galvanic vestibular stimulation

The extracellular neural recording in the vestibular nucleus (VN) was performed based on our previous studies^25,14,20^. The neural recording was initiated by defining a center on the skull. Generally, the center was positioned at 3.0 mm posterior from the lambda, and the VN was positioned at 3.0 mm posterior and 2.0 mm lateral away from the center. Once the recording position was defined, an animal was anesthetized by injecting the solution (see Parkinson’s Disease model), and its head was fixed in the motorized stereotaxic apparatus (NEUROSTAR, Germany). Then, the animal’s skull was surgically exposed, and a recording hole (2–2.5 mm diameter) was opened. Through the hole, a recording electrode (5MΩ, A-M system, US) was inserted to search a neuronal response, and all neuronal responses were continuously tested using kinetic head movements, such as horizontal head rotation and linear head transmission. The relation between the neuronal responses to the head movement, such as head rotation, head linear transmission or both, was used to confirm that the recorded activities were those of the vestibular neurons. Once the vestibular responses were confirmed based on the responses to the kinetic stimuli, galvanic vestibular stimulation (GVS) was applied. GVS (100 μA) was applied through the stimulation electrode located between the temporal muscles and bones, and the electrodes were placed at both left and right sides. Their corresponding polarities were positive and negative, respectively. The neuronal signals were filtered (bandpass 0.5–3 kHz) and recorded (sampling rate: 40 kHz) using OmniPlex D system (Plexon, US).

### Analysis of neuronal signals

The neuronal signals were analyzed based on the instantaneous firing rates (IFR), which were the reciprocal number of inter-spike interval (ISI). Their comparison was made by the averaged IFR for 10-s resting period. To specify the toxic (6-OHDA) effects, the neurons were grouped as the canal- and the otolith-related units, and each group was again subdivided into regular and irregular responses, depending on the neuronal discharge regularity. The discharge regularity was calculated using the normalized coefficient of variance (CV*), and its computational function was as like below;

1 $$CV^{*} = 10 \times \left[ {\frac{CV}{{0.7116\log \left( \mu \right) - 0.8248}}} \right]^{{{\raise0.7ex\hbox{$1$} \!\mathord{\left/ {\vphantom {1 {\left( {0.00002\mu^{3} - 0.0024\mu^{2} + 0.0731\mu + 0.37} \right)}}}\right.\kern-\nulldelimiterspace} \!\lower0.7ex\hbox{${\left( {0.00002\mu^{3} - 0.0024\mu^{2} + 0.0731\mu + 0.37} \right)}$}}}}$$

where µ is the mean of ISI and CV is the coefficient of variance. Based on the calculated CV*, all neurons were divided into regular and irregular units. For a statistical comparison, two-sample t-test was applied. By comparing two sub-groups in four different groups (canal & regular, canal & irregular, otolith & regular, and otolith & irregular), the toxic effects were examined.

### Immunohistochemistry (IHC) & assessment of glutamate receptors

The effects of GVS were assessed by the change in the number of glutamate receptors, focusing on α-amino-3-hydroxyl-5-methyl-4-isoxazole-propionic acid (AMPA) and N-methyl-D-aspartate (NMDA) receptors. The overall process was similar as prepared in staining brain tissues (see Model Evaluation) by applying the antibodies of the glutamate receptors. In summary, all animals’ brains were removed after completing stimulation, and resulted in five groups (control, PD model, PD model with 100 μA, PD model with 500 μA, and PD model with 1000 μA). To provide the designed amount of GVS, all animals involved in the assessment of glutamate receptors underwent GVS-only under anesthesia. The methods of stimulation and anesthesia were similar as indicated in the extracellular neuronal recording (see Extracellular neural recording & Galvanic Vestibular Stimulation). To assess the effects of GVS in PD models, different amounts of GVS were applied. A set of GVS was composed of 20-time stimuli with the same stimulating duration (3 s) and frequency (once every 60 s). A 10-min interval was provided between two stimulating sets, and total three sets of GVS were applied with different amplitudes (100, 500, and 1000 μA). Based on the given conditions of GVS, the relevant electrical charges were 0.018, 0.09, and 0.18 coulombs for 100, 500, and 1000 μA, repectively. The electrical charge was calculated as follows;

2 $$q = 3 \times \mathop \sum \limits_{1}^{20} \mathop \smallint \limits_{{t_{1} }}^{{t_{2} }} I\left( t \right)dt$$

where, *q* was the total electrical charge, and *I(t)* was the current of GVS. The interval between *t*_*1*_ and *t*_*2*_ was the stimulating duration.

The brains from five groups were prepared for the immunohistochemistry of AMPA and NMDA receptors. In 4% formaldehyde solution, the portion of VN was fixated and paraffinized. The prepared paraffin block was sliced by 2.5–3 µm, and the selected slices were used for antibody staining. Using GluR1 (#ABN241, Sigma-Aldrich, US) and NR1 (#G8913, Sigma-Aldrich, US), AMPAr and NMDAr were stained. The prepared slices were rinsed, and their endogenous peroxidase activity was blocked (3% hydrogen peroxide solution). Then, specific antigen retrieval solutions were applied (Tris–EDTA, pH 9.0, 95 °C, 50 min & citrate solution, pH 6.0, 95 °C, 50 min) for each subtype, and stored at 4 °C overnight. Staining the subtypes of receptors, their images were taken by a motorized microscope (Olympus, Tokyo, Japan) with a magnification of 400 times (ocular: × 10 and objective: × 40). The specific location of VN was confirmed in the images, and the number of receptor positive cells was manually counted with several independent repetition.

### Statistical analysis

All statistical significances in Figs. 3 and 6 were assessed by two-sample t-test (MATLAB, MathWorks, Boston, USA). Using a given function, the means of two independent populations were examined if they were equal or not.

## Data Availability

The supporting data for the current findings in this study are available from the corresponding author upon reasonable request.
